# Global Trends in Research of Amino Acid Metabolism in T Lymphocytes in Recent 15 Years: A Bibliometric Analysis

**DOI:** 10.1155/jimr/3393342

**Published:** 2025-01-25

**Authors:** Jiaona Xu, Yinan Yu, Shijie Li, Fanghui Qiu

**Affiliations:** ^1^Department of Rehabilitation, Hangzhou Geriatric Hospital, Hangzhou 310022, China; ^2^Department of Rehabilitation, Affiliated Hangzhou First People's Hospital, Westlake University School of Medicine, Hangzhou 310006, China; ^3^Department of General Surgery, Sir Run-Run Shaw Hospital, Zhejiang University School of Medicine, Hangzhou 310018, China

**Keywords:** amino acid metabolism, bibliometric, citespace, T lymphocytes, VOSviewer

## Abstract

Amino acid metabolism in T cells determines the therapeutic efficacy of T-cell-targeting drugs. To assess the direction of amino acid metabolism in T cells and construct related knowledge structure, we performed a bibliometric analysis aiming at amino acid metabolism in T cells utilizing studies publicized in recent 15 years. Three hundred thirty-seven related studies were downloaded from the Web of Science Core Collection (WoSCC), and the information on countries, institutes, and authors was collected and analyzed. In addition, the present research status and future trends were explored according to the results yielded from the analysis of cited references and keywords. This study revealed that publications regarding amino acid metabolism in T cells gradually increased each year. The USA is the top producer and most influential country in this field. Recent research has focused on the correlation between the metabolism of several amino acids and regulatory T cells (Tregs) and CD8^+^ T cells. Overall, this research offers a comprehensive exhibition on the field of amino acid metabolism in T cells, which will help researchers to study this domain more effectively and intuitively.

## 1. Introduction

T lymphocytes are the primary components of lymphocytes that originate from bone marrow pluripotent stem cells and play an essential role in cell-mediated immunity. Currently, T-cell-targeting drugs are exhibiting positive outcomes in the treatment of various diseases [1–3]. However, the efficacy of immunotherapy is heavily reliant on the longevity, differentiation, and functionality of T cells, which are largely determined by the metabolic activity of T cells [4, 5]. Thus, the efficacy of immunotherapy can be amplified by modulating the metabolic conditions of T cells [6].

Amino acids are essential for the biosynthesis of polyamines, nucleotides, glucosamines, and antioxidants. They also function as metabolites that generate energy after entering the energy production process [7]. Transporting amino acids across the plasma membrane is typically mediated by the solute carrier (SLC) family, and over 60 SLC members have been identified as amino acid transporters to date [8]. These identified transporters can be subdivided into neutral, basic, and acidic classes based on their substrate, and all three classes can be classified into sodium ion (Na^+^) dependent or independent [9]. Amino acids are vital nutrients for immunological cells, particularly T lymphocytes. T cell proliferation, differentiation, and activation are heavily reliant on amino acid metabolism, as well as intracellular calcium ion (Ca^2+^) concentration fluctuations [10–12]. For instance, activated T cells utilize alanine for protein synthesis, and alanine deficiency inhibits both memory and naive T cell activation [13, 14]. In addition, increased methionine uptake upon T cell activation helps to maintain RNA and histone methylation [15, 16]; and methionine deprivation causes reduced histone H3K4 methylation (H3K4me3) in Th17 cells [17] and H3K79me2 in CD8^+^ T cells [16], indicating that methionine is an essential source for T cell function and survival. These findings demonstrate the importance of amino acid metabolism in maintaining T cell homeostasis and the immunotherapeutic efficacy of drugs that target amino acid metabolism. Notably, recent trials have produced compelling evidence that the amino acid metabolism of immune cells changed after immunotherapy, thereby, affecting the therapeutic efficacy [10, 18]. For example, methionine supplementation restores H3K79me2 and STAT5 function and expression in CD8^+^ T cells in colon cancer patients, suggesting the value of avoiding methionine deficiency in T cells to provide clinical value [16].

To date, amino acid metabolism in T cells has attracted considerable attention from various research fields. However, there are significant gaps in our understanding of particular amino acids and their SLCs on the phenotype and function of various types of T cells [9], meaning that studies on amino acid metabolism in T cells are still in the developmental stage. Analyzing what has been accomplished in this field in depth will help us predict the developmental trend in amino acid metabolism in T cells and provide new ideas for basic research and clinical prevention and treatment.

Bibliometrics is an approach to library and information science based on the quantitative analysis of the bibliographic content [19]. Recently, bibliometric analysis has gained great popularity in medical research and its popularity can be attributed to its utility in processing large amounts of scientific data and exerting high research influence. Bibliometric analysis can provide scholars with new trends in article and journal performance, collaborative patterns, research components, and knowledge structures in specific areas in existing literature [20]. Owing to the application of bibliometrics over the past years, the process of studying T cells has been greatly accelerated [21, 22]. However, bibliometric analysis on the correlation between amino acid metabolism and T cells is still void.

Thus, we here conducted a bibliometrics analysis exploring hotspots and development trends of amino acid metabolism in T cells based on studies published in recent 15 years. Furthermore, we summarized the basic and clinical aspects of the popular research directions obtained from the analysis results. The study aims to help more scientists in related areas gain a better understanding of the past, present, and future of amino acid metabolism in T cells, leading to more efficient and effective exploration of the frontiers, hotspots, and trends of this field.

## 2. Materials and Methods

### 2.1. Data Sources and Search Strategies

**Figure 1:**
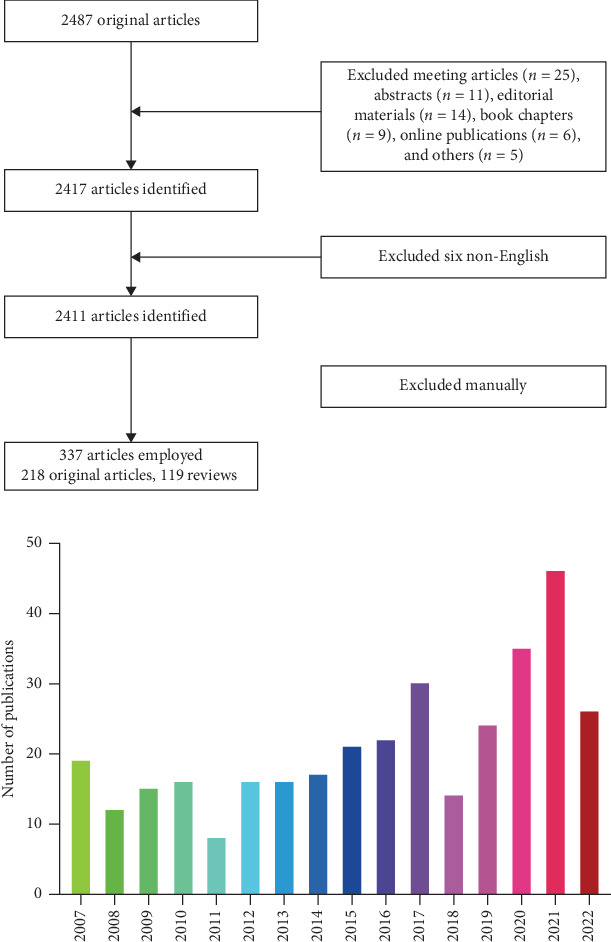
(A) Flowchart of the screening process. (B) Overall distribution of publication outputs on amino acid metabolism in T cells: global annual output trends.

The Web of Science Core Collection (WoSCC) was used to search and obtain information on amino acid metabolism in T cells in recent 15 years (from 2007 to 2022). Literature retrieval was performed within 1 day (December 31, 2022) to avoid fluctuations in citations attributed to the rapid updates of publications. The topics we mainly focus on are the effect of amino acid intake from diet, secreted by other cells in the microenvironment, or generated by T cells themselves on the fate of T cells. The search formula was set to TS = (“amino acid”) AND TS = (“T lymphocytes” OR “T cells”). A total of 2487 studies were filtered out in this step. Next, only reviews and original articles were enrolled for analysis, and 78 articles including meeting articles (*n* = 25), editorial materials (*n* = 14), book chapter (*n* = 9), online publication (*n* = 6), meeting abstract (*n* = 11), and others (*n* = 5) were excluded. Moreover, only articles written in English remained (*n* = 2411). Finally, the title, abstract, or even full text of these researches were read to screen out papers closely associated with the topic we studied here. Finally, only 337 publications were included in this bibliometrics analysis.  Figure 1A depicts the procedure in detail. The following information was extracted from these studies, which are the number of publications and citations in each year, the names, countries, and affiliations of the authors, journals, references, and keywords. This procedure was performed by three researchers independently (Shijie Li, Jiaona Xu, and Yinan Yu) and any potential differences were discussed. The publications list of the 337 articles is provided in the Supporting Information.

### 2.2. Statistical Analysis

All valid data were imported to Microsoft Office Excel 2019, HistCite (version 2009.08.24), VOSviewer (version 1.6.18), Bibliometrix 4.1.0 Packages based on the R language, and CiteSpace (version 5.8.R2) to perform visual analysis. Microsoft Office Excel 2019 was hired to plot radar charts.

HistCite [23] was utilized to determine the number of publications, the total local citation score (TLCS), and the total global citation score (TGCS) for each publishing year, as well as the leading nations, authors, journals, and institutions.

VOSviewer [24] was contracted to visualize the bibliometric network, encompassing country and institution cooperation. The sizes of the nodes indicate the number of publications, while the thickness of the lines indicates the strength of the relationship.

CiteSpace [25] was utilized to undertake cluster analysis, reference bursts, keyword searches, and timeline views. By categorizing keywords and references, cluster analysis of significant terms can uncover crucial aspects of amino acid metabolism in T cells. The modularity *Q* > 0.3 and mean silhouette >0.5 suggested that the findings of clustering are sufficient and compelling. Keyword and reference bursts can be utilized to identify emerging research trends in amino acid metabolism in T cells by analyzing keyword and reference spikes.

The Bibliometrix Packages [26] is a tool based on the R language used for bibliometric analysis, which was used to analyze the annual growth rate of publications and the top 10 cited references here.

## 3. Results

### 3.1. The Trend of Publication Outputs

Based on the search strategy, a total of 218 original studies and 119 reviews on amino acid metabolism in T cells were screened out. The number of articles published during each period reflects the development trend of research in this field. As is shown in Figure 1B, the number of articles gradually increased each year (annual growth rate: 2.11%). Notably, the number of publications in 2020 and 2021 is the highest in the past 15 years. In light of this, it is worthwhile to research and investigate amino acid metabolism in T cells as it is currently a hot topic.

### 3.2. Analysis of Countries/Regions

**Figure 2:**
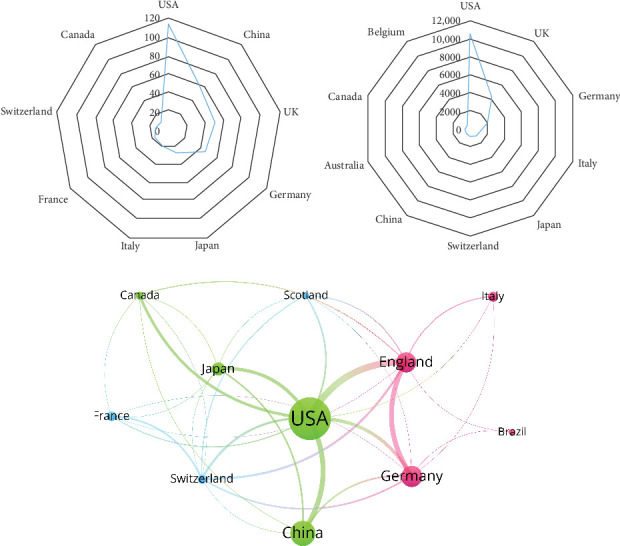
Analysis of countries/regions. (A) Radar map of the top 10 productive countries. (B) Radar map of total global citation score (TGCS) of the top 10 productive countries. (C) Visual cluster analysis of cooperation among countries. The size of each circle indicates the number of articles produced by that country. The distance between two circles represents their coauthorship relationship, while the thickness of the connecting line indicates the strength of the relationship.

From 2007 to 2022, 30 countries conducted research on amino acid metabolism in T cells.  Table 1 displays the 10 countries with the largest number of outputs. Over the past 15 years, the United States has published the most studies on amino acid metabolism in T cells, with a total of 113. China (*n* = 56) and the United Kingdom (*n* = 51) ranked second and third, respectively (Figure 2A).

The United States is the most cited country for published research (10,707 citations), followed by the United Kingdom (4222 citations) and Germany (2020 citations; Figure 2B). A further coauthorship network comprising countries/regions with at least five publications was established. The network indicated that collaboration between the displayed countries is relatively close. The United States has cooperated with nearly all other nations, and England has the closest relationship with the United States (Figure 2C).

### 3.3. Analysis of Institutions and Authors

**Figure 3:**
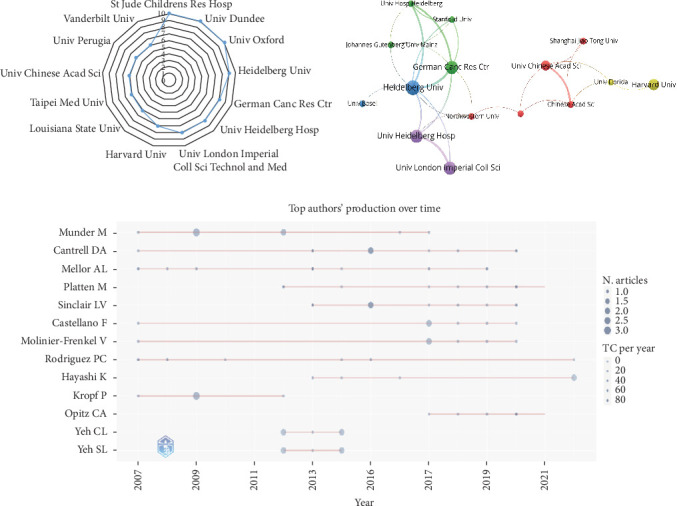
Analysis of institutions and authors. (A) Radar map of the top 13 productive institutions. (B) Visual cluster analysis of cooperation among institutions. The size of each circle indicates the number of articles produced by that institution. The distance between any two circles indicates the relatedness of their coauthorship link, and the thickness of the connecting line indicates the strength of the link. (C) The top authors' production over time.

A total of 611 institutions have researched T cell amino acid metabolism.  Table 2 lists the 13 institutions with the most publications. St Jude Children's Research Hospital (*n* = 10) in the United States, the University of Dundee (*n* = 10) and the University of Oxford (*n* = 10) in the United Kingdom, and Heidelberg University in Germany (*n* = 9) were the leading institutions in terms of outputs (Figure 3A). Notably, Louisiana State University (*n* = 9) is the most cited institution, indicating the highest influence on the field of amino acid metabolism in T cells. The institutions with greater than or equal to four publications were used to construct the coauthorship network. The network indicated that cooperation among the given institutions is rather close; however, it appears that this cooperation may be increased. (Figure 3B).

A total of 2169 scholars have written articles on amino acid metabolism in T cells to date, and Table 3 lists the 13 most prolific authors. Among them, Munder M was the most productive author with eight publications, followed by Cantrell DA (seven papers), Mellor AL (seven papers), Platten M (seven papers), and Sinclair LV (seven papers). Mellor AL is the most cited author (1923 times), followed by Munn DH (1445 times) and Rodriguez PC (1290 times), which shows that these three authors have a strong influence on the work of other authors and institutions. In addition, a timeline of writers who have published studies on amino acid metabolism in T cells was created (Figure 3C). Cantrell DA, Mellor AL, Castellano F, Molinier-Frenkel V, and Rodriguez PC have been interested in this field for more than a decade since 2007; Munder M has been interested in this field for approximately 10 years since 2007; Kropf P has only published papers between 2007 and 2012; the remaining six authors have been interested in amino acid metabolism in T cells after 2012.

### 3.4. Analysis of Journals

A total of 192 journals accepted studies on amino acid metabolism in T cells, and Table 4 lists the top 10 core journals. There was a total of 70 articles published in these 10 journals. Frontiers in Immunology was the most prolific journal, publishing 18 papers, followed by Journal of Immunology (14 articles) and European Journal of Immunology (seven articles). Cancer Research was the most referenced journal with 1243 citations, followed by Journal of Immunology (1135 times) and Blood (1000 times).

### 3.5. Analysis of Cited and Cocited References

**Figure 4:**
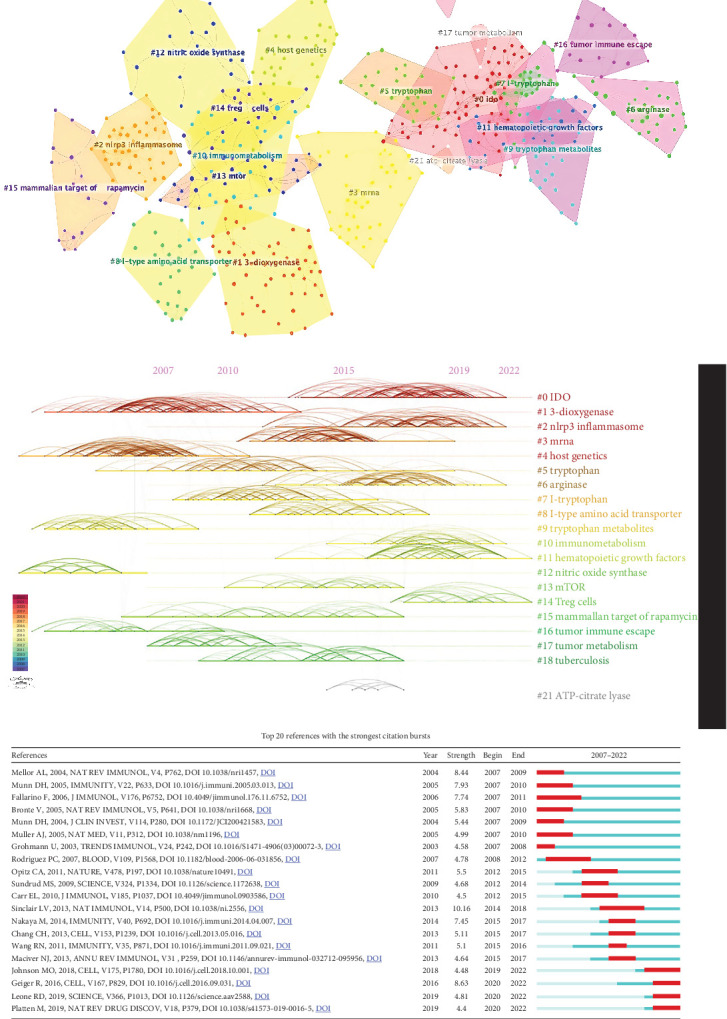
Analysis of cited and cocited references. (A) Cluster analysis of cocited references. (B) Timeline distribution of the top 20 subject term clusters in the corresponding cocited articles. (C) Top 20 references with the strongest citation bursts. The red line on the time axis indicates the period during which the respective article was cited.

Cited and cocited references are both the basis for researchers who concentrate on amino acid metabolism in T cells. Therefore, we analyzed both cited and cocited studies. The top 10 cited researches among the 337 objects and the top nine cocited references are presented in Tables 5 and 6, respectively. The study reported by Munn et al. [27] is the most cited and the fourth cocited reference, in which GCN2 was identified as a molecular sensor of indoleamine-2,3-dioxygenase (IDO), an enzyme that catalyzes tryptophan (Trp), in T cells, enabling these cells to detect and react to conditions created by IDO. Besides, the research emphasizing the importance of L-type amino acid transporter in T cell differentiation ranks second in citation and first in cocitation, which is published by Sinclair et al. [28]. Moreover, a cluster analysis on the ground of cocited references was performed here, and the value of modularity *Q* and mean silhouette were 0.8628 and 0.945, respectively, indicating the excellent quality of this analysis. Twenty clusters that meet the requirement of *K* values were plotted (Figure 4A), which contain “IDO,” “Trp,” “arginase,” “regulatory T cells (Tregs),” “tumor metabolism,” et cetera [28]. Specially, four of the 20 clusters are associated with Trp, highlighting the vital role of Trp in T cells. Moreover, a timeline was plotted based on these clusters to show the transformation of these clusters with time (Figure 4B). Relatively, L-type amino acid transporter, a Na^+^ independent transporter, is newly concerned to researchers. Finally, the top 20 references with the strongest citation burst are exhibited in Figure 4C. Among them, studies focus on L-Arginine (L-Arg; reported by Geiger et al. [29]), glutamine (Gln; reported by Leone et al. [30]), and Trp (reported by Platten et al. [31]) possess relatively high citation bursts from 2020 to 2022, indicating the popularity of these three amino acids recently.

### 3.6. Analysis of Keywords

**Figure 5:**
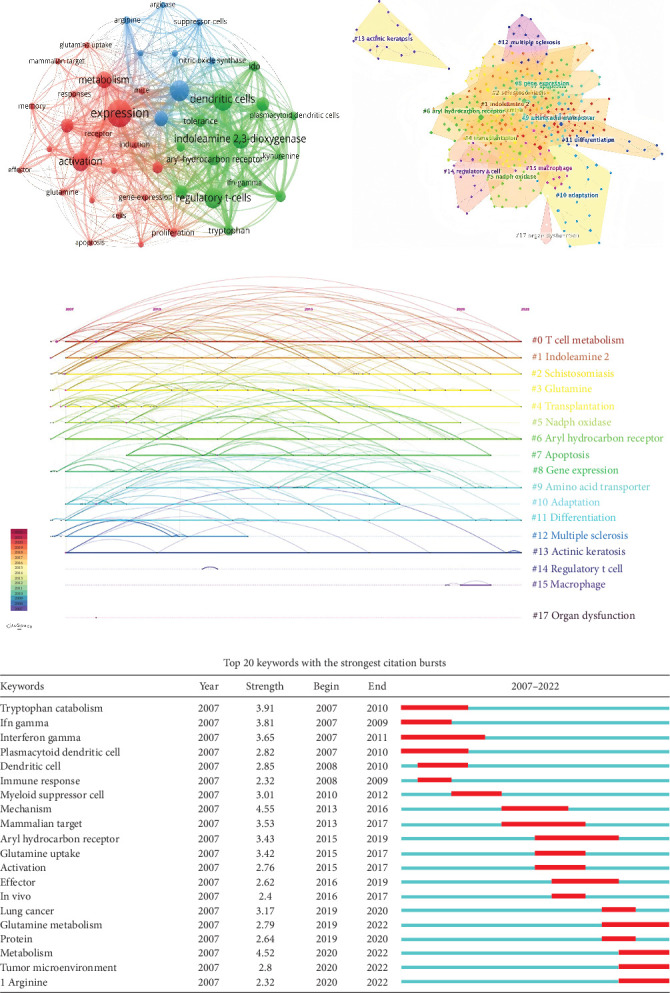
Analysis of keywords. (A) Conetwork of keywords. The size of each circle indicates the number of occurrences of the corresponding keyword. The distance between any two circles indicates the keywords' link, and the thickness of the connecting line indicates the strength of the link. (B) Cluster analysis of keywords. (C) Timeline distribution of the top 17 clusters of keywords. (D) Top 20 keywords with the strongest citation bursts. The red line on the time axis indicates the period of bursts.

We first built a network based on the keywords extracted from enrolled documents (Figure 5A). For T cell types, we found the circle representing Tregs is the largest. Furthermore, we found the existence of IFN-*y*, a cytokine secreted by CD8^+^ T cells. These two cues indicated amino acid metabolism in Tregs and CD8^+^ T cells draw large attention in recent 15 years. Besides T cells, we surprisingly found that the scale of the circle representing dendritic cells is obvious, suggesting that amino acids may act as a bridge connecting dendritic cells and T cells. In simple terms, the alteration of amino acids in dendritic cells affects the ability of dendritic cells to present antigens to T cells, and as a result, the activation of T cells is tuned [32]. Besides T cell types, three amino acids named Gln, kynurenine (Kyn), and Arg are also popular in recent 15 years. Further, we constructed a keywords cluster analysis and displayed the top 17 clusters (Figure 5B). The modularity *Q* and mean silhouette value of this analysis were 0.7477 and 0.8903, respectively. According to the clusters obtained, we subsequently plotted a timeline (Figure 5C). The clusters obtained confirmed the popularity of Tregs and Gln. Additionally, the popularity of IDO was highlighted in these two analyses, suggesting the potential for therapeutic regimens targeting key enzymes of amino acid metabolism. Finally, the top 20 keywords with the strongest are shown in Figure 5D. The results indicated that the effect of Gln and Arg in T cells in diseases, especially in tumor microenvironment (TME), draw the attention of researchers in the latest years.

## 4. Discussion

In this study, we investigated the primary knowledge domain and emerging trends pertaining to amino acid metabolism in T cells. The annual publishing output has increased over time while maintaining a relatively high number of citations. The United States was the most productive of the 30 countries, with the highest proportion of the top 13 productivity institutions. However, among the 30 researchers we studied, the most prolific author was Munder M. from Germany. His team mainly focused on the effect of Arg metabolism on T lymphocyte proliferation and function [33–39]. Their most recent T cell research reveals unexpected characteristics of T cell activation under Arg deprivation and demonstrates the potential of citrulline as a precursor amino acid for T cell endogenous Arg synthesis and subsequent departure from the immunosuppressed state [33]. Furthermore, they discovered that reducing L-Arg induces autophagy in human T cells as a cytoprotective response to endoplasmic reticulum stress, providing a deeper understanding of the regulation of T cell survival or death [35]. Among the top 13 most productive institutions, the most cited institution is the Louisiana State University in the United States (published six articles and cited 1838 times). The most cited publication at Louisiana State University was published in 2007 by Rodriguez, Quiceno, and Ochoa [40], who discovered that L-Arg availability regulates T lymphocyte cell-cycle progression. Specifically, they reported that impaired T cell proliferation caused by L-Arg deficiency was associated with the inability to upregulate the expression of cyclin D3 and cdk4, but not cyclin D1, cyclin D2, or cdk6.

**Figure 6:**
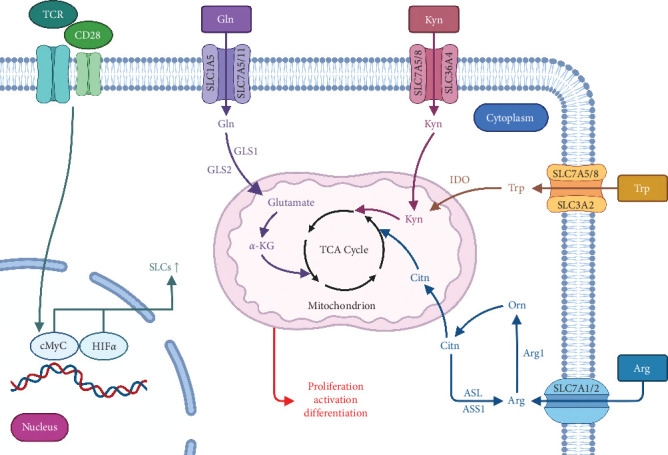
Roles of amino acids (Gln, Trp, Kyn, and Arg) and their transporters in T cell function. TCR engagement and costimulation affect multiple pathways that are mainly involved in cMyc and HIF1*α*, and upregulate amino acids (Gln, Trp, Kyn, and Arg) transporters, SLCs in T cells, thereby increasing their uptake. As a result, these amino acids enter T cells via their respective transporters SLCs, are metabolized according to the depicted pathway, and then enter the TCA cycle to support T cell activation, proliferation, and differentiation. *α*-KG, *α*-ketoglutarate; Arg, arginine; Arg1, arginase-1; ASL, argininosuccinate lyase; ASS1, argininosuccinate synthase 1; Citn, citrulline; Gln, glutamine; GLS1/2, glutaminase 1/2; IDO, indoleamine-2,3-dioxygenase; Kyn, kynurenine; Orn, ornithine; SLC, solute carrier; TCA cycle, tricarboxylic acid cycle; TCR, T cell receptor; Trp, tryptophan.

Recent studies indicate that amino acid metabolism regulates the fate of CD4^+^ T cells [34, 41, 42]. Moreover, based on the timelines of references and keywords, as well as the conetwork or burst of keywords, our findings demonstrated the strong association between amino acids (such as Gln, Trp, Kyn, and Arg) and CD4^+^ T cells, particularly Tregs (Figure 6). Amino acid metabolism plays a crucial role in Tregs development, facilitating their adaptation within the microenvironments [43]. During naïve T cells stimulation, limiting Gln in the media or adding the glutaminase inhibitor 6-diazo-5-oxo-l-norleucine (DON) increased FoxP3 expression in vitro in a TGF-*β*-dependent manner. Such FoxP3^+^ Tregs generated under Gln restriction maintain immune system suppression and can survive in vivo microenvironments [44, 45]. On the contrary, Gln limitation will suppress Th1 and Th17 differentiation [11, 44]. Unlike Gln, high-level glutamate can directly change Tregs and boost their proliferation and suppressive activity [46, 47]. T cell receptor (TCR) activation and costimulation influence several pathways, including the upregulation of SLCs, and the amino acid transporters in Tregs, which increases Gln absorption [9]. Additional glutamate converted by Gln in Tregs can then directly alter them [46, 47]. Similarly, in the TME, tumor cells convert Gln to glutamate, which can be exported in exchange for cystine, leaving the TME low in Gln and high in glutamate, which may support tumor-infiltrating Tregs (TI-Tregs) [48–50]. Furthermore, researchers are interested in the significance of T cell amino acid metabolism in the context of the COVID-19 pandemic. It is demonstrated that decreased intracellular Gln levels result in decreased *α*-ketoglutarate (*α*-KG) levels, which stimulate the development of Treg cells. This is consistent with a broad spectrum of immunosuppression in COVID-19 and is associated with poor outcomes for patients with severe COVID-19 [51, 52].

Moreover, based on our data, IDO is a key enzyme in amino acid metabolism that has recently drawn great attention. It could mediate the conversion of Trp to Kyn [53]. IDO-mediated Trp depletion and the resulting Trp metabolites promote the development of FoxP3^+^ Tregs and activate the suppressive activity of Tregs in a dendritic cell-dependent manner [54–57]. Kyn is known to activate and signal through the aryl hydrocarbon receptor (AHR), which is essential for TGF-*β* dependent activation of Tregs [58, 59]. The overexpression of IDO in melanoma tumors led to an increase in the expression of AHR on TI-Treg and an improvement in their suppressive capabilities [60]. In addition, tissue occupant Tregs and activated Tregs in peripheral circulation can deplete Arg due to increased Arg expression and use this pathway to limit effector T cell proliferation. These Tregs with elevated Arg expression were also seen in melanoma tumors [61]. These findings suggest that Tregs can exploit the amino acid patterns of their microenvironment to activate, survive, and function.

In addition to CD4^+^ T cells, amino acids are also indispensable to CD8^+^ T cells (Figure 6). As indicated by “IFN-*γ*” in the conetwork of keywords we analyzed, further studies are required to examine the effect of amino acid metabolism on the killing power of CD8^+^ T lymphocytes. Geiger et al. [29] discovered that Arg supplementation stimulates the development of central memory-like T cells with high survival capacity, hence, boosting the antitumor activity of CD8^+^ T cells in melanoma cells. Moreover, after COVID-19 infection, the body's Arg activity increases, resulting in Arg depletion, which further inhibits signal transduction of TCRs and IFN-*γ* production, ultimately resulting in T cell malfunction and exacerbating inflammation [62, 63]. Kyn can be directly transported into CD8^+^ T cells via SLC7A5/8 and SLC36A4 to activate AHR and upregulate PD-1 expression, which causes the depletion of CD8^+^ T cells [64, 65]. Therefore, inhibiting the Kyn–AHR pathway increases the antitumor effectiveness of CD8^+^ T cells. Trp metabolism is closely related to the Kyn–AHR pathway. Trp is catalyzed by IDO or Trp-2,3-dioxygenase (TDO) to become Kyn [8]. The Kyn pathway is presumed to break down more than 95% of free Trp in CD8^+^ T lymphocytes [66]. Thus, Trp depletion and Trp–Kyn–AHR-related metabolites contribute to CD8^+^ T cell malfunction and tumor immune evasion. Similarly, Trp and its metabolites can be controlled to variable degrees in COVID-19 disease, resulting in an “exhaustion” state of CD8^+^ T lymphocytes mediated by the AHR, which slows or hinders their capacity to destroy virus-infected cells. This process has also become a major predictor of COVID-19 infection severity and mortality [67, 68]. For Gln metabolism, recent investigations demonstrated that Gln limitation could increase the anticancer efficacy of TI CD8^+^ T cells [69]. However, activation of memory CD8^+^ T cells, which increases TCR downstream signaling, enhances T cell-mediated cytotoxicity, and promotes effector cytokine release, is contingent on a specific Gln concentration [70]. Given the significance of amino acid metabolism on T cell activation, drugs or inhibitors targeting the major amino acids and their essential enzymes or transporters in the aforementioned metabolic pathways are crucial for the treatment of diseases, especially cancers.  Table 7 lists medications that target amino acid metabolism in T cells.

In general, extensive data supports the determinant effect of both the intracellular and extracellular amino acids on T cell fate [82]. The field has shifted its initial focus from amino acids themselves in T cell metabolism to the mechanisms of their acquisition and transport and is also investigating their connections with other biological processes [83–85]. With the expansion of research in this area, numerous metabolic intervention methods and medications are in development. However, recent failures in clinical trials necessitate additional research in this area. For instance, how are T cells able to outcompete other cell types for essential amino acids? How can the intervention window elicit a persistent anticancer effect be determined [86]? As only a few of the 60 SLCs in T cells have been studied in the mouse model, basic research must also consider the influence of individual amino acids and their corresponding SLCs. Future studies should require an in-depth understanding of these amino acids and SLCs in T cells in the knockout mouse model, which will provide us with us more visual insights into therapeutic strategies based on T cell amino acid metabolism [8, 9].

We investigated the historical and future developments in the field of amino acid metabolism in T cells using a comprehensive bibliometrics analysis. However, there are still numerous limitations. First, despite our best efforts to collect the most comprehensive literature, some papers were omitted from the study, potentially introducing bias. Second, due to the short time frame, some high-quality research published in recent years may have received insufficient citations; these studies were not highlighted in this analysis. This study employs an algorithm, which may result in marginally insufficient evidence.

## 5. Conclusion

In conclusion, our results indicate that studies into amino acid metabolism in T cells are currently developing rapidly. The USA is the major producer, resulting in numerous breakthroughs in this field. Among the research institutions, Louisiana State University is the institution with the highest influence on achievements. Mellor AL is an outstanding contributor to the field of amino acid metabolism in T cells. Frontiers in Immunology, Journal of Immunology, Cancer Research, and Blood were the most prolific and/or cited journals that published the latest studies and innovative developments. Finally, the correlation between the metabolism of amino acids (such as Gln, Trp, Kyn, and Arg) and Tregs and CD8^+^ T cells has recently received increased attention, which will also be the focus of future research.

## Figures and Tables

**Table 1:** The top 10 productive countries concerning amino acid metabolism in T cells.

Rank	Country	Counts	TLCS	TGCS
1	USA	113 (33.5%)	392	10,707
2	China	56 (16.6%)	10	695
3	UK	51 (15.1%)	180	4222
4	Germany	45 (13.4%)	71	2020
5	Japan	24 (7.1%)	46	1062
6	Italy	17 (5%)	34	1172
7	France	16 (4.7%)	18	413
8	Switzerland	14 (4.2%)	25	797
9	Canada	12 (3.6%)	27	525
10	Brazil	10 (3%)	4	171

Abbreviations: TGCS, total global citation score; TLCS, total local citation score.

**Table 2:** The top 13 productive institutions concerning amino acid metabolism in T cells.

Rank	Institution	Counts	TLCS	TGCS
1	St Jude Children's Research Hospital	10 (3%)	13	522
2	University of Dundee	10 (3%)	66	1063
3	University of Oxford	10 (3%)	27	811
4	Heidelberg University	9 (2.7%)	15	328
5	German Cancer Research Center	8 (2.4%)	15	695
6	Heidelberg University Hospital	8 (2.4%)	37	830
7	Univ London Imperial Coll Sci Technol and Med	8 (2.4%)	35	456
8	Harvard University	7 (2.1%)	23	1026
9	Louisiana State University	6 (1.8%)	84	1838
10	Taipei Med University	6 (1.8%)	2	79
11	University of Chinese Academy of Sciences	6 (1.8%)	4	150
12	University of Perugia	6 (1.8%)	29	605
13	Vanderbilt University	6 (1.8%)	1	118

Abbreviations: TGCS, total global citation score; TLCS, total local citation score.

**Table 3:** The top 13 productive authors concerning amino acid metabolism in T cells.

Rank	Author	Counts	TLCS	TGCS
1	Munder	8	34	462
2	Cantrell	7	66	986
3	Mellor	7	82	1923
4	Platten	7	15	653
5	Sinclair	7	65	910
6	Castellano	6	13	176
7	Molinier-Frenkel	6	13	176
8	Rodriguez	6	69	1290
9	Hayashi	5	17	106
10	Kropf	5	26	345
11	Opitz	5	4	175
12	Yeh	5	2	69
13	Yeh	5	2	69

Abbreviations: TGCS, total global citation score; TLCS, total local citation score.

**Table 4:** The top 10 core journals publishing amino acid metabolism in T cells.

Rank	Journal	Counts	TLCS	TGCS
1	Frontiers in Immunology	18 (5.3%)	0	542
2	Journal of Immunology	14 (4.2%)	76	1135
3	European Journal of Immunology	7 (2.1%)	31	410
4	Frontiers in Oncology	7 (2.1%)	0	130
5	Cancer Research	6 (1.8%)	41	1243
6	Nature Communications	6 (1.8%)	0	775
7	PLoS ONE	6 (1.8%)	0	151
8	Proceedings of the National Academy of Sciences of the United States of America	6 (1.8%)	44	580
9	Blood	5 (1.5%)	68	1077
10	Oncoimmunology	5 (1.5%)	0	146

Abbreviations: TGCS, total global citation score; TLCS, total local citation score.

**Table 5:** The top 10 cited references among the 337 publications.

Rank	First author	Year	Journal	DOI
1	Munn	2005	Immunity	https://doi.org/10.1016/j.immuni.2005.03.013
2	Sinclair	2013	Nature Immunology	https://doi.org/10.1038/ni.2556
3	Mellor	2004	Nature Review Immunology	https://doi.org/10.1038/nri1457
4	Munn	1998	Science	https://doi.org/10.1126/science.281.5380.1191
5	Fallarino	2006	Journal of Immunology	https://doi.org/10.4049/jimmunol.176.11.6752
6	Uyttenhove	2003	Nature Medicine	https://doi.org/10.1038/nm934
7	Wang	2011	Immunity	https://doi.org/10.1016/j.immuni.2011.09.021
8	Rodriguez	2007	Blood	https://doi.org/10.1182/blood-2006-06-031856
9	Munn	1999	Journal of Experimental Medicine	https://doi.org/10.1084/jem.189.9.1363
10	Nakaya	2014	Immunity	https://doi.org/10.1016/j.immuni.2014.04.007

**Table 6:** The top nine cocited publications in amino acid metabolism in T cells.

Rank	First author	Year	Journal	DOI
1	Sinclair	2013	Nature Immunology	https://doi.org/10.1038/ni.2556
2	Geiger	2016	Cell	https://doi.org/10.1016/j.cell.2016.09.031
3	Fallarino	2006	Journal Immunology	https://doi.org/10.4049/jimmunol.176.11.6752
4	Munn	2005	Immunity	https://doi.org/10.1016/j.immuni.2005.03.013
5	Mellor	2004	Nature Review Immunology	https://doi.org/10.1038/nri1457
6	Nakaya	2014	Immunity	https://doi.org/10.1016/j.immuni.2014.04.007
7	Bronte	2005	Nature Review Immunology	https://doi.org/10.1038/nri1668
8	Muller	2005	Nature Medicine	https://doi.org/10.1038/nm1196
9	Mondanelli	2017	Immunity	https://doi.org/10.1016/j.immuni.2017.01.005

**Table 7:** Main chemical compounds target amino acid metabolism in Tregs/CD8^+^ T cells.

Chemical compounds	Target	Main effect on T cells	Related diseases	References
ADI-PEG20	Arg (amino acid)	Tregs ↓ CD8^+^ T ↑	Mesothelioma; HCC; PDAC; AML	[71–74]
CB-1158	Arg1 (key enzymes of Arg)	CD8^+^ T ↑	Advanced or metastatic solid tumors	[9, 75]
Epacadostat	IDO1 (key enzyme of Trp)	Tregs ↓ CD8^+^ T ↑	Unresectable or metastatic melanoma; advanced solid tumors	[76, 77]
Navoximod			Advanced solid tumors; cervical tumors;	[78, 79]
IDB-AHRi	AHR (key target of Kyn)	Tregs ↓ CD8^+^ T ↑	Colon cancer	[9]
PEG-KYNase	Kyn (amino acid)	Tregs ↓ TI-CD8+ T ↑	Melanoma; breast carcinoma; colon carcinoma	[80]
SLC7A5 mAb	SLC7A5 (key transporter of Kyn)	Tregs ↓ CD8^+^ T ↑	Colon cancer	[81]

Abbreviations: ↑, increase; ↓, decrease; AHR, aryl hydrocarbon receptor; AML, acute myeloid leukemia; Arg, arginine; Arg1, arginase-1; HCC, hepatocellular carcinoma; IDO1, indoleamine-2,3-dioxygenase 1; Kyn, kynurenine; PDAC, pancreatic ductal adenocarcinoma; SLC, solute carrier; TI-CD8^+^ T, tumor-infiltrating CD8^+^ T cells; Tregs, regulatory T cells; Trp, tryptophan.

## Data Availability

The data that supports the findings of this study are available in the Supporting Information of this article.

## Supporting Information

Supporting Information Supporting Information Description: Details of the 337 articles analyzed in this paper.
