# Erratum to “Global Trends in Research of Amino Acid Metabolism in T Lymphocytes in Recent 15 Years: A Bibliometric Analysis”

**DOI:** 10.1155/jimr/9813495

**Published:** 2025-06-24

J. Xu, Y. Yu, S. Li, and F. Qiu, “Global Trends in Research of Amino Acid Metabolism in T Lymphocytes in Recent 15 Years: A Bibliometric Analysis,” *Journal of Immunology Research* 2025, no. 1 (2025): 1–16, https://doi.org/10.1155/jimr/3393342.

In the article titled “Global Trends in Research of Amino Acid Metabolism in T Lymphocytes in Recent 15 Years: A Bibliometric Analysis,” an affiliation was omitted in error. The corrected author list and affiliation list should be as follows:

Jiaona Xu^1,2^, Yinan Yu^3^, Shijie Li^4^, Fanghui Qiu^1,2^

^1^Department of Rehabilitation, Hangzhou Geriatric Hospital, Hangzhou 310022, China

^2^Department of Rehabilitation, Affiliated Hangzhou First People's Hospital, Westlake University School of Medicine, Hangzhou 310006, China

^3^Department of Oncology, Shaoxing People's Hospital, Shaoxing, Zhejiang Province, Shaoxing 312300, China

^4^Department of General Surgery, Sir Run-Run Shaw Hospital, Zhejiang University School of Medicine, Hangzhou 310018, China

We apologize for this error.

